# Polydomy in the ant *Ectatomma opaciventre*

**DOI:** 10.1093/jis/14.1.21

**Published:** 2014-01-01

**Authors:** Viviane C. Tofolo, Edilberto Giannotti, Erika F. Neves, Luis H. C. Andrade, Sandro M. Lima, Yzel R. Súarez, William F. Antonialli-Junior

**Affiliations:** 1 Universidade Estadual Paulista “Júlio de Mesquita Filho”, Instituto de Biociências, Centro de estudos de Insetos Sociais (CEIS), Rio Claro/SP, Brazil; 2 Universidade Estadual Paulista “Júlio de Mesquita Filho”, Instituto de Biociências, Departamento de Zoologia, Rio Claro/SP, Brazil; 3 Universidade Federal da Grande Dourados, Dourados, Programa de pós-graduação em Entomologia e Con-servação da Biodiversidade, MS, Brazil; 4 Universidade Estadual de Mato Grosso do Sul (UEMS), Centro Integrado de Análise e Monitoramento Ambiental, Dourados/MS, Brazil

**Keywords:** colony organization, cuticular hydrocarbons, nestmate recognition, satellite nests, spatial distribution

## Abstract

Tropical ants commonly exhibit a hyper-dispersed pattern of spatial distribution of nests. In polydomous species, nests may be satellites, that is, secondary structures of the main nest, where the queen is found. In order to evaluate whether the ant *Ectatomma opaciventre* Roger (Formicidae: Ectatomminae) uses the strategy of building polydomous nests, the spatial distribution pattern of 33 nests in a 1,800 m ^2^ degraded area located in Rio Claro, SP, Brazil, were investigated using the nearest neighbor method. To complement the results of this investigation, the cuticular chemical profile of eight colonies was analyzed using Fourier transform infrared photoacoustic spectrosco-py (FTIR-PAS). The nests of *E. opaciventre* presented a hyper-dispersed or regular distribution, which is the most common in ants. The analysis of the cuticular hydrocarbons apparently con-firmed the hypothesis that this species is polydomous, since the chemical profiles of all studied colonies with nests at different sites were very similar to the chemical signature of the single found queen and were also different from those of colonies used as control.

## Introduction

Ants of the genus *Ectatomma* belong to the family Ectatomminae ( Bolton et al. 2007 ) and are endemic to the Neotropical Region ( Brown Jr 1958 ; Lattke 1994 , 2003). Among the 14 species of the genus, 10 can be found in Brazil: *E. brunneum, E. edentatum, E. lugens, E. muticum, E. opaciventre, E. permagnum, E. planidens, E. ruidum, E. suzanae* , and *E. tuberculatum* ( Kugler and Brown 1982 ; Oliveira and Brandão 1991 ; Santos et al. 1999 ; Silvestre and Silva 2001; Marinho et al. 2002 ; Franz and Wcislo 2003 ; Corrêa et al. 2006 ; Marques and Del-Claro 2006 ; Delabie et al. 2007 ; Vieira et al. 2007 ; Scott-Santos et al. 2008 ).

Studies done on this genus deal mainly with its interactions with plants that have extrafloral nectaries ( Oliveira and Brandão 1991 ; Oliveira and Freitas 2004 ; Oliveira and Del-Claro 2005 ), fauna surveys ( Marinho et al. 2002 ; Marques and Del-Claro 2006 ), ecology ( Silvestre and Silva 2001 ; Pie 2004 ; Erdogmus 2010; Tofolo et al. 2006 , 2010, 2011), population dynamics ( Antonialli-Junior and Giannotti 1997 , 2001; Tofolo and Giannotti 2005 ; Vieira et al. 2009 , 2010), division of labor ( Antonialli-Junior and Giannotti 2002 ; Vieira et al. 2010 ), nest architecture (Antoni-alli-Junior and Giannotti 1997; Lapola et al. 2003 ; Vieira et al. 2007 ), and chemical ecology ( Antonialli-Junior et al. 2007 , 2008).

For most social insects, colonies are organized with the presence of a queen, workers, and brood, and the physical architecture of nests varies according to the group (Höldobler and Wilson 1990). In ants, due to the large number of living species ( Agosti and Johnson 2005 ), this variation occurs at the species level (Höldobler and Wilson 1990). In *Ectatomma* , for example, the architecture of nests and population dynamics meet the general standards of the group, such as simple architecture and a small number of adults, as well as the behaviors and habits of life (lack of recruitment between workers, predominantly carnivorous habits, and age polyethism). However, according to the species, small variations are found, such as the number of chambers (2 to 10), the nest maximum depth (68 cm to 360 cm), the average number of adults (22 to 94) and, more intriguingly, the presence or absence of fertilized queens in the nests excavated. In *E. brunneum* , *E. opaciventre* , *E. edentatum* , and *E. vizottoi* , varying amounts of virgin queens were found; however, fertilized queens were not present in all nests excavated, although all of them contained immature individuals ( Paiva and Brandão 1989 ; Antonialli-Junior and Giannotti 1997 , 2001; Lapola et al. 2003 ; Vieira and Antonialli-Junior 2006 ; Vieira et al. 2007 ; Tofolo et al. 2010). In cases where nests were kept in the laboratory after collection, workers emerged from the field pupae; over time, this caste developed ovaries and began oviposition, resulting only in males until the end of their life cycle ( Lapola et al. 2003 ; Tofolo et al. 2010 ).

The difficulty in finding queens in this ant genus is be supported by the theory of polydomy. According to Hölldobler and Wilson (1990) , a polydomous nest is a central structure (which houses workers, queens, and brood) with interconnecting secondary units, called satellite nests, containing a portion of workers and sometimes brood. Seifert (2010) considers that polydomy implies the existence of specific features, such as polygyny, reduced size of males and gynes, intranidal mating or short-range nuptial flights, sharing of the food source, exchange of individuals and brood between nests, and the development of a system of recognition among nestmates. Anderson and McShea (2001) and (2011) also consider that the limits to which the nest belongs are not restricted only to the physical structure that houses the queen, workers, and immatures, but also involve adaptive changes in the environment explored by the colony, the so-called outspots ( Hölldobler and Wilson 1990 ) and outstations (Anderson and Mcshea 2001). These structures include shelters dug into the ground that foragers use to rest for an extended period or, in the case of photophobic ants, to protect themselves temporarily from sunlight, as in *Formica integra* ( Step 1924 ), *Lasius fuligi-nosus* , *L. niger, F. pratensis* , (Dobrzañska 1966), *Oecophylla* sp. ( Hölldobler and Wilson 1977 ), *Eurhopalothrix heliscata* ( Wilson and Brown 1984 ), and *Crematogaster torosa* ( Lanan et al. 2011 ).

Polydomy, therefore, is not restricted to the number of queens present in the secondary units, as it occurs in both mono-and polygynous species) ( Laskis and Tschinkel 2009 ). It would be a strategy (temporary or permanent) adopted by some ant species to avoid space constraints that would occur if the entire colony remained only in the central nest ( Jaffé 1993 ). It also allows for a more efficient exploitation of resources dispersed in the environment ( Laskis and Tschinkel 2009 ), although it may hinder the distribution of resources, social regulation, and communication between the subunits created ( Crozier and Pamilo 1996 ).

In *Ectatomma* , satellite nests are known to be an inherent feature of Ectatomminae ( Hölldobler and Wilson 1990 ); however, until now, no experiment has in fact proven that it is associated with polydomy. During the excavation of the nests used in studies with *E. brunneum* ( Lapola et al. 2003 ; Tofolo and Giannotti 2005 ; Locher et al. 2009 ; Tofolo et al. 2010 ), *E. opaciventre* ( Antonialli-Junior and Giannotti 1997 ; Tofolo et al. 2011 ), *E. planidens* ( Antonialli-Junior and Giannotti 2000 , 2001, 2002, 2003; Antonialli-Junior et al. 2007 ), and *E. vizottoi* ( Vieira et al. 2009 ), it was noticed that not all of them had queens, as some had only workers and brood. Furthermore, no underground or surface structure delimiting trails and connecting nests nearby has been found so far, as occurs in species that build outstations.

Although *E. opaciventre* do not have sophisti-cated recruitment methods, communication between the subunits created is made possible with the use chemical cues ( Hölldobler and Wilson 1990 ), maintaining colony cohesion and preventing the exploitation of its resources by invaders ( Crozier and Pamilo 1996 ). This recognition occurs through a mixture of chemical compounds present on the cuticle of individuals called surface pheromones, which are both genetically and environmentally determined. They are largely composed of saturated and unsaturated hydrocarbons ( Blomquist et al. 1998 ; Lenoir et al. 1999 ; Blomquist and Bagnères 2010 ). These cuticular hydrocarbons play a particularly important role in nestmate recognition because they are the most abundant compounds on the cuticle of ants and often contain clues used to distinguish between nestmates and non-nestmates ( Provost 1989 ; Singer 1998 ; Sturgis and Gordon 2012 ). They tend to be speciesspecific, although they may vary depending on the species, colony, caste, diet, age, and environmental conditions ( Howard 1993 ; Howard and Blomquist 2005 ; Blomquist and Bagnères 2010 ).

According to Crozier and Dix (1979) , nestmates must keep contact on a regular basis to effectively maintain the ability to tolerate their nestmates. Nevertheless, this contact is hampered when satellite nests are concerned because individuals do not keep continuous contact with one another, which may produce a unique chemical label within each nest, causing a mosaic of odors ( Dahbi and Lenoir 1998 ). According to Bonavitta-Cougourdan et al. (1987) and Meunier et al. (2011) , workers memorize specific proportions of these chemical substances, which enables them to detect and reject any intruder (workers or eggs) that presents quantitative differences in their chemical profiles. This behavior may be recorded by differences in the level of aggression between workers of different nests (Nowbahari et al. 1990).

The investigation of polydomy in *E. opaciventre* may reveal part of its natural history and determine what the spatial and colonial limits of the species are, that is, how individuals use space and how the colony is distributed and organized regarding the different structures that compose the nest. This type of information can be the basis for further studies on behavior, reproduction, and evolution. Proving the existence of subgroups within a social species reveals different complexities in the relationship between individuals of a colony.

In this work, a combination of physical data (location and structure of colonies) and chemical data (cuticular chemical profiles) was used to explore and understand how the spatial organization of colonies of *E. opaciventre* Roger (Formicidae: Ectatomminae) takes place. Recently this subfamily was classified into the Poneroid clade for presenting more basal features, and more recently Brady et al. (2006) and Moreau (2006) relocated this subfamily into the Formicoid clade, along with the most derived species. The existence of polydomous nests in this species is one of the main questions to be answered, given the difficulty of collecting queens between nests nearby and the absence of trails connecting the subunits built.

## Methods

### Study species and location

*Ectatomma opaciventre* is an ant species endemic to the Neotropical Region ( Brown Jr 1958 ), as in northern Argentina and southeastern Venezuela and also in areas of cerrado and caatinga in central Brazil ( Kugler and Brown 1982 ). It presents carnivorous habits (Fernán-dez 1991). The workers forage individually in the epigeal stratum in the daytime period ( Pie 2004 ), collecting mainly living or dead individuals of Hymenoptera, Coleoptera, Lepidoptera, and Orthoptera ( Tofolo et al. 2011 ). Unlike other *Ectatomma* , no liquid food, such as honeydew or nectar, was observed ( Erdogmus 2010 ). They exhibit foraging area fidelity and employ individual foraging strategies, i.e., there is no recruitment of nestmates for food search or transport ( Pie 2004 ).

*Ectatomma opaciventre* nests in the hypogeal stratum ( Fernández 1991 ) and builds vertical nests at depths of up to 68 cm. The architecture of these nests is simple, with a single chimney-like entrance cavity of 0.9 ± 0.2 cm in diameter and 1.6 ± 0.6 cm high, which gives access to a maximum of five chambers. However, the inner walls of the chambers and the tunnels connecting them are coated with tightly-packed soil because of the composition of the salivary secretion of these ants (Antoni-alli-Junior and Giannotti 1997). Thus, the entire nest structure is clearly visible during excavation, allowing the exploration of all chambers. Their colonies can present from nine to just over 100 individuals in the dry and rainy seasons, respectively ( Antonialli-Junior and Giannotti 1997 ; Tofolo 2011 ).

The spatial distribution of the ant nests may be considered regular or hyper-dispersed, as a result of the competition for food sources between colonies ( Traniello 1989 ) or the predation of founding queens by the oldest colonies, which prevents new colonies from settling ( Ryti and Case 1986 ; Hölldobler and Wilson 1990 ; Deslippe and Savolainen 1995 ). Random or clumped distributions may occur as a result of interspecific interactions (Levings and Traniello 1981; Ryti and Case 1984 , 1986; Hölldobler and Wilson 1990 ). In nests of *E. opaciventre* in preserved sites, Pie (2004) found a hyper-dispersed pattern of spatial distribution, the most common in this kind of area.

The active nests of *E. opaciventre* were located in a degraded area of 1,800 m ^2^ (designated as area A) in the city of Rio Claro, SP, Brazil (22º 22’ 49.73” S; 47º 33’ 48.87” W). This area is located in the urban region and is therefore surrounded by homes. It is predominantly covered by grasses and constantly suffers the action of burning and brush cutters. The area presents no other shrubby or woody vegetation, so the landscape has little heterogeneity and the richness of species of ants and other arthropods is low.

Among the nests found in area A, eight were chosen for collection because their foraging workers showed higher activity. Because the nests were vertical, with one chamber built below the other, the collection started 30 cm away from the only entrance hole, where a round trench was excavated to a depth of 50 to 100 cm. This way, the resulting cylinder of soil contained the entire nest. With the aid of a spatula, the excavation was then carried out laterally, moving inwards, until each chamber was reached one by one and all the individuals in them were collected. The individuals of each colony were transferred to the laboratory in artificial plaster nests containing three chambers of increasing sizes, as in Antonialli-Junior and Giannotti (1997) . The ants were fed every day for three months with a source of protein (larvae of *Tenebrio molitor* L. (Coleoptera: Tenebrionidae)), carbohydrate (sugar-water solution 1:1), and water until they were used in the experiments for extraction of cuticular hydrocarbons.

### Nest location spatial distribution pattern

In order to analyze the pattern of spatial distribution of nests of *E. opaciventre* , the spatial position of each nest was recorded using a tape measure and then plotted as X and Y coordinates on a chart. The average distance between them and the pattern of spatial distribution were calculated by means of the nearest-neighbor method by Clark and Evans (1954) . According to this method, an aggregation index (R) equal to 1 means a random distribution pattern; if it approaches zero, an aggregated pattern; if it is higher than 1, with an upper limit of 2.12, it is a hyper-dispersed pattern.

### Fourier transform infrared-photoacoustic spectroscopy (FTIR-PAS)

To identify the similarity between the cuticular chemical profiles of individuals of eight colonies in area A, 15 workers (n = 120), one gyne, and the single queen were stored in 70% alcohol. As a control, 15 workers and one queen collected in another similar area (designated as area B, 22º 22’ 49.44” S; 47º 33’ 02.08” W, approximately 1 km away from area A) were used.

The gaster was extracted from the ants and placed in a vacuum oven for 48 hr to minimize moisture absorption, which would be undesirable in this study. Infrared beams of light were directed at the gaster of the ants because this is a part of the body that presents high concentrations of cuticular hydrocarbons and it is handled easily ( Cuvillier-Hot et al. 2001 ). The equipment used was a Thermo-Nicolet Nexus 670 (Thermo Scientific, www.thermoscientific.com ) spectrophotometer coupled to a MTEC-300 photoacoustic detector (MTEC, www.mtecpas.com ); the data were processed using Omnic software (Thermo Scientific) supplied by the equipment manufacturer, following the report by Antonialli-Junior et al. (2007 , 2008).

The degree of similarity between individuals of different colonies was evaluated by the intensity of the peaks obtained via FTIR-PAS. To confirm the results, the data went through a principal component analysis (PCA). The Euclidean distances between the colonies sampled were correlated with spatial position using a Mantel test (999 permutations) to quantify the influence of spatial proximity in FTIR-PAS similarity ( Valentin 2000 ).

## Results

### Nest spatial distribution

**Figure 1.**
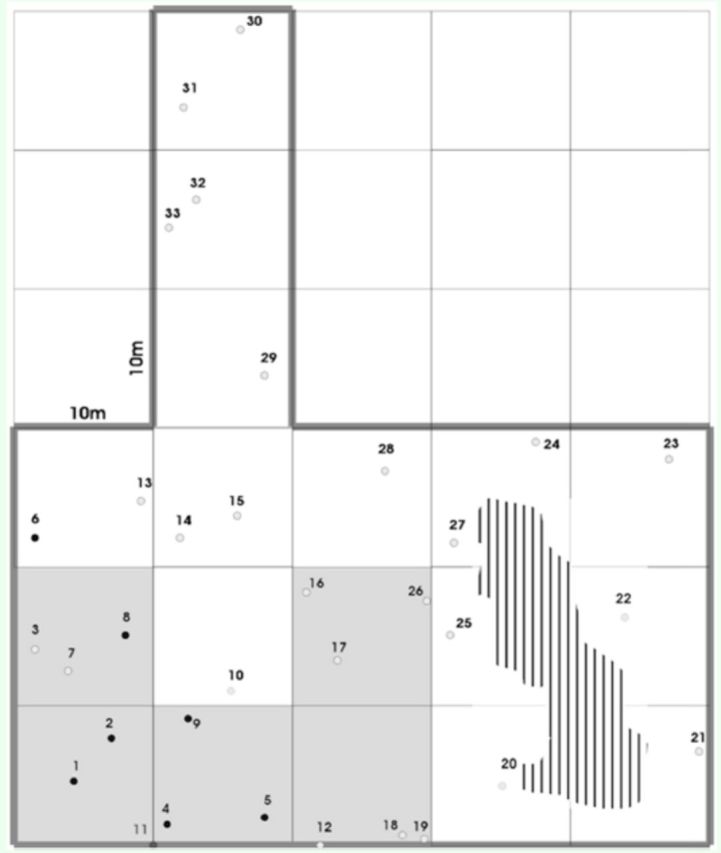
Map of the 33 nests of *Ectatomma opaciventre* foundin a 1,800 m2 area. The study area A is delimited by a gray line,and the hatched area corresponds to a waste dump. The blackcircles indicate the nests collected. The single queen was foundin nest 11.

A total of 33 active colonies of *E. opaciventre* were located, and the nest density in the area ( Figure 1 ) was 0.018 nests/m ^2^ . According to Clark-Evans (1954) method, the pattern of spatial distribution of the nests is regular or hyper-dispersed (R = 1.51), with an average distance between nests of 5.6 ± 0.4 m (ranging from 1.93 m to 12.96 m).

The population data from the nesting colonies in area A show that only one nest had a queen and, in this nest, the number of larvae of different instars was relatively higher than in the other groups ( Table 1 ). The average number of chambers found was 4 ± 1; the highest number was six and the smallest was three. None of the nests collected had eggs or pupae.

**Table 1.**
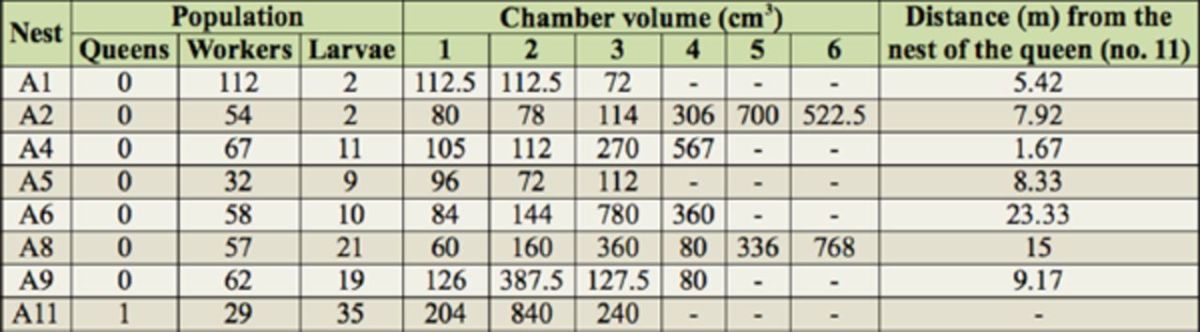
Populational and physical data of the eight nests of *Ectatomma opaciventre* collected in area A in the city of Rio Claro,SP, Brazil.

No trails, surfaces, or underground structures connecting these nests were found. There was no record of aggressive behavior between foragers at trail intersections.

### FTIR-PAS

**Figure 2.**
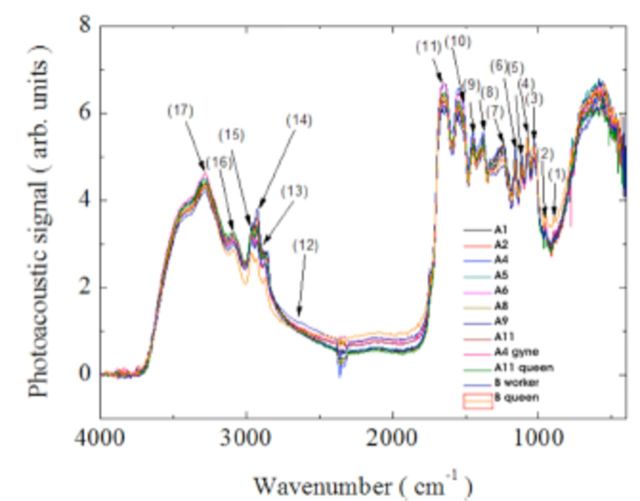
Spectra of the cuticular hydrocarbons extracted from adults of eight colonies of *Ectatomma opaciventre*

The analysis of the cuticular hydrocarbons of ants of the eight most active colonies indicated 17 mid-infrared peaks ( Figure 2 ) with superior absorption; they were considered the most important to separate the groups. Table 2 shows the functional groups and the vibrational mode of each of the peaks previously described by Antonialli-Junior et al. (2007 , 2008).

**Table 2.**
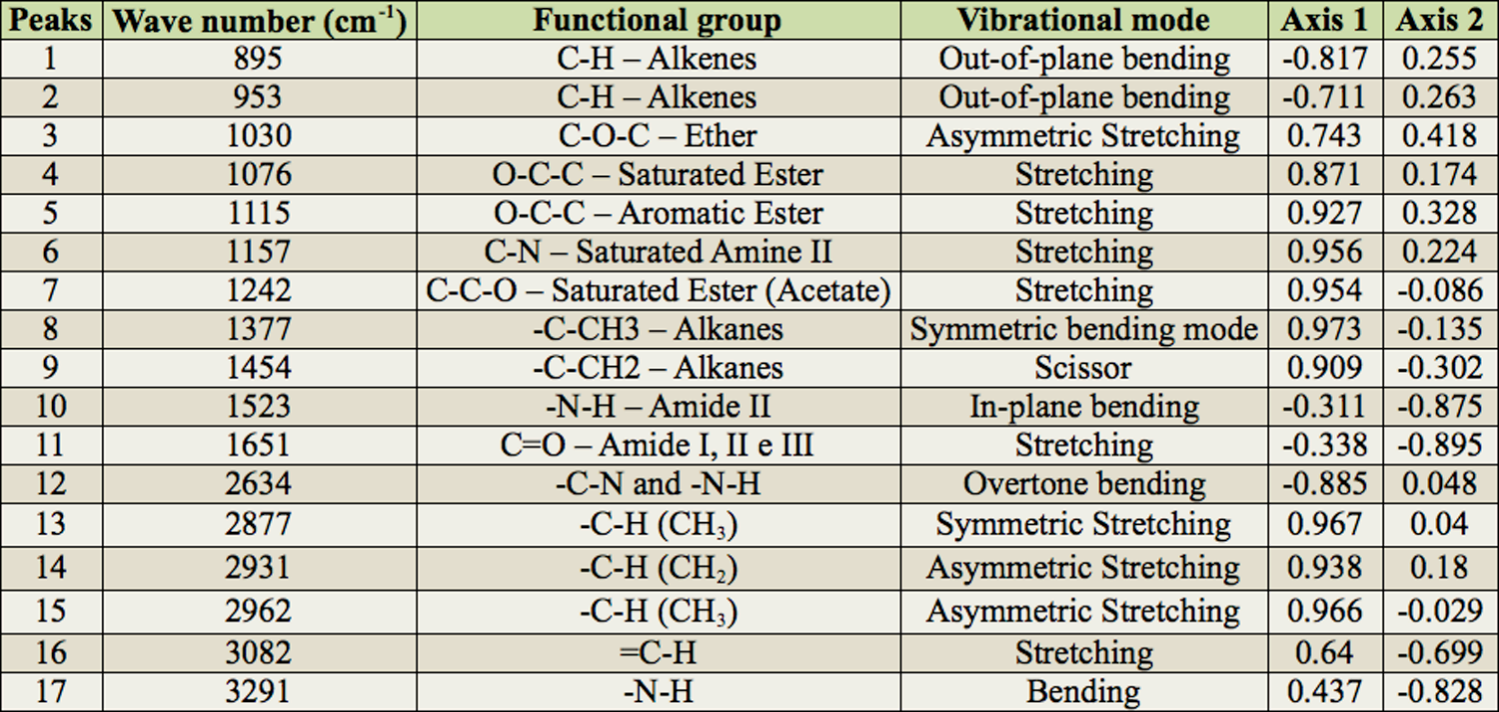
Representation of the photoacoustic peaks of higher vibration corresponding to the extraction of cuticular hydrocarbons from the abdomen of workers of eight colonies of *Ectatomma opaciventre* and loadings of each peak on the first two axes of the PCA results.

**Figure 3.**
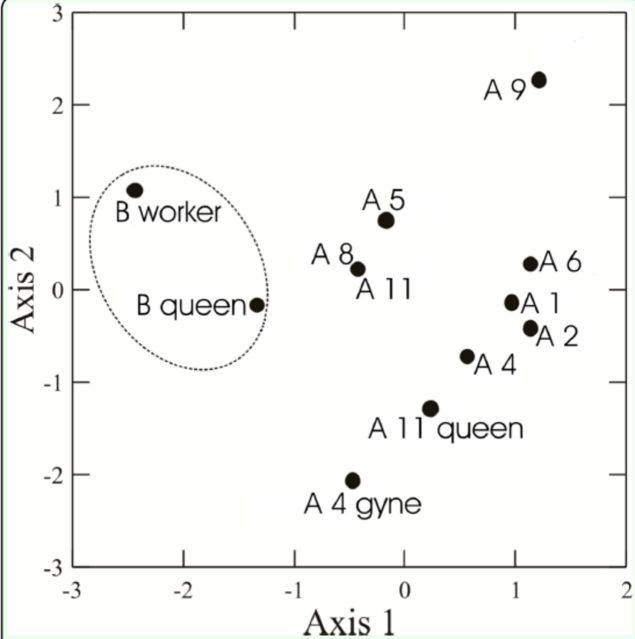
Scatterplot of principal component analysis based on the profiles of the cuticular hydrocarbons from different coloniesof the ant *Ectatomma opacivente* : A 1, A 2, A 4, A 4 gyne, A5, A 6, A 8, A 9, A 11, and A 11 queen correspond to the samplesof adults collected from nests of area A; B queen and Bworker correspond to adults collected from area B (controlarea)

The PCA analysis showed that the profiles of cuticular hydrocarbons of queens, workers, and gynes were different from one other quantitatively. The data of the colonies evaluated in area A overlapped more, and therefore there was a greater similarity among them than among those in area B ( Figure 3 ). Thus, it was possible to distinguish between at least two groups, one composed of the data of the profiles of cuticular hydrocarbons of queens, gynes, and workers of eight colonies of area A, and the other consisting of the control samples in area B ( Figure 3 ).

Considering the loadings of the FTIR-PAS peaks in the first axis, the A group had higher intensity of peaks 895 and 2634 cm ^-1^ , while the control group presented smaller intensity of these peaks and higher intensity of many other peaks, such as 1377, 2877, and 2962 cm In the second axis, the peaks 1523, 1651, and 3290 cm ^-1^ had higher intensity in the gyne and queen of the A group ( Table 2 ). The Mantel test showed that there was a significant correlation between nest distances and the chemical profiles of their cuticular hydrocarbons (r = 0.64; *p* = 0.01). Thus, colonies near each other presented more similar cuticular hydrocarbon signatures.

## Discussion

The spatial distribution pattern of the nests of this species was regular or hyper-dispersed, the most common pattern in ants ( Levings and Traniello 1981 ), such as *Aphaenogaster seni-lis* , *Messor barbarus* , *Solenopsis latro* , *Tetramorium semilaeve* , *Tapinoma nigerrima* , and *Plagiolepis pygmaea* ( Redolfi et al. 2005 ). This probably occurs because all these species are territorial and, upon increasing nest density, the competition between them also increases ( Greenslade 1975 ; Ryti and Case 1984 , 1986; Cushman et al. 1988 ). In fact, when nests of *E. opaciventre* were studied in a preserved area, Pie (2004) reported the same hyper-dispersed pattern of spatial distribution, with a density smaller than that found in the current study. This was an expected result, considering that the diversity and resources available to species are higher in a preserved area, resulting in greater spacing between nests. This difference was clearly observed by Deslippe and Savolainen (1994) in nests of *Formica podzolica* located in forest edges with high resource availability and in meadows.

Other studies showed that nest density in ants of the genus *Ectatomma* varies widely, unlike the pattern of spatial distribution, which in most cases is also hyper-dispersed, as in *E. ruidum* ( Breed et al. 1990 ; Pratt 1989 ), *E. permagnun* ( Paiva and Brandão 1989 ), and *E. tuberculatum* ( Wheeler 1986 ). Probably, *E. opaciventre* established unicoloniality in the study area, as there was a complete absence of hostility between occupants from different nests ( Crozier and Pamilo 1996 ), even when foraging trails overlapped.

The results of our study demonstrate that there are strong indications that this species has polydomy, because among the colonies evaluated, only one had a queen and it contained a greater number of immatures ( Table 1 ). Moreover, the analysis of the profiles of cuticular hydrocarbons shows that most of these colonies had, in fact, greater similarity between them than those considered as controls, and that many of the profiles of cuticular hydrocarbons of workers from different colonies were close to that of the queen of colony 11. The large distance between the data regarding colonies 5, 8, and 9 in area A and the data concerning the remaining colonies suggests that they may be part of another set of polydomous nests.

Mantel’s test confirms that the smaller the distance between them, the greater the similarity of their profiles of cuticular hydrocarbons, indicating the possibility that the individuals evaluated might come from eggs laid by a single queen, i.e., that these individuals could belong to a single colony.

Although interaction tests between ants from different nesting sites were not performed, in numerous personal observations workers were caught entering and leaving nests near each other, carrying other workers and immatures. Such behavior was not uncommon during the excavations.

Further evidence should be taken into account. In previous work with this species, a relatively high proportion of colonies collected in the field had no queens ( Antonialli-Junior and Giannotti 1997 ). In addition, other species such as *E. brunneum* present the same evidence as well. Lapola et al. (2003) and Vieira and Antonialli (2007) also suggest polydomy for this species. In *E. tuberculatum* , Zinck et al. (2008) proved polydomy based on the responses to the recognition mechanisms between workers of the same nest and of different nests.

Considering that all colonies analyzed were raised in a laboratory after collection, and that the larvae developed into new workers, it becomes more evident that these workers may be daughters of the same queen, since non-fecundated workers could not produce females, but only males. In fact, Antonialli-Junior and Giannotti (1997) observed workers of *E. opaciventre* laying trophic eggs and eggs that gave rise to males, but never to other females.

In fact, the hyper-dispersed pattern of spatial distribution and the building of structures secondary to the main nest (satellites and outstations) seem to be an alternative to the energy expenditure required during environment exploration. Colony division, for example, allows a decrease in the foraging costs of the central nest so that the secondary units increase the dispersion area of the species and resource collection ( Debout et al. 2007 ). The concentration of workers in various subunits over space may also allow quick recruitment when necessary ( Anderson and McShea 2011 ).

According to a definition by Forel (1974), polydomy is characterized by several nests with the absence of brood or queens in at least one of them; they grow following a strategy that minimizes problems with space ( Hölldobler and Wilson 1990 ) and temperature ( Pedersen and Boomsma 1999 ) and reproductive conflict with the queen ( Banschbach and Herbers 1996 ). In the case of the colonies of *E. opaciventre* collected in our study, all of them had workers and brood and only one of them had a queen, since it is a monogynous species ( Table 1 ). The nests without a queen may be regarded as satellites of the central nest, forming a polydomous structure.

Therefore, the chemical identity of the workers from colony 11 was expected to approach that of the queen from same colony. The level of similarity between the workers belonging to the other nests in area A was not the same between all of them. This variability is also expected, considering that each nest has a specific microenvironment and that the formation of cuticular hydrocarbons depends not only on genetic factors (heritable cues), but may also be environmentally influenced ( Howard and Blomquist 2005 ; van Zweden and D’ettorre 2010 ). It is important to acknowledge that these local differences identified them as a group different from that of area B (control area).

Nonetheless, it is worth considering that the genetic similarity between the colonies of area A may also be explained by the degree of relatedness between them, due to the limited dispersal of the colonies. In this case, they should all present queens, which was not the case. It is still possible that the absence of queens was due to some problem during excavation and collection. However, careful measures were taken so that these problems were minimized. Moreover, other studies report the absence of queens after collection of colonies of the same species ( Antonialli-Junior and Giannotti 1997 ) and other species of the genus ( Lapola et al. 2003 ; Vieira and Antonialli 2007 ).

On the other hand, it is known that the profile of cuticular hydrocarbons may be influenced by environmental factors, such as the incorporation of hydrocarbons from food sources and nesting material. Some studies have shown that the differences in the chemical profiles of individuals from different nests remain for a short time ( Jutsum et al. 1979 ; Liang and Silverman 2000 ), with a predominance of the genetically determined composition ( Bagnères and Blomquist 2010 ). Once individuals or colonies are subjected to the same biotic and abiotic conditions, it becomes much more likely that the predominant chemical profile is more genetically than environmentally determined ( Howard and Blomquist 2005 ). The results found for the composition of the cuticular chemical profile of the ants did not suffer or suffered little environmental and food influence, as the ants used were not subjected to the chemical analysis immediately after collection in the field but were taken to the laboratory and held for more than three months under the same biotic/abiotic conditions.

### Conclusion

*Ectatomma opaciventre* presents hyper-dispersed spatial distribution of nests, as do most species of tropical regions. As already discussed in other works, both this form of spatial distribution of nests and polydomy may be strategies to reduce intra-and interspecific competition. Polydomy could be a strategy to allow species dispersion, minimiz-ing foraging costs and diluting the effects of predation.

In this study, evidence was found that this species may have polydomous nests, because most of the colonies showed no queens, only workers and immatures. The chemical analysis of these colonies showed that those closest to one another had similar profiles of cuticular hydrocarbons, especially compared to the only colony in which a queen was found. These findings thus reinforce the hypothesis formu-lated, based both on the populations presented by each colony and on laboratory and field observations.

## Abbreviations

FTIR-PAS: Fourier transform infrared photoacoustic spectroscopy

## References

[R1] Agosti D Johnson NF . Editors. 2005 . Antbase. American Museum of Natural History . Available online: http://www.antbase.org

[R2] Anderson C McShea DW . 2011 . Intermediate-level parts in insect societies: adaptive structures that ants build away from the nest . Insectes Sociaux 48 : 291 – 301 .

[R3] Antonialli-Junior WF Giannotti E . 1997 . Nest architecture and population dynamics of *Ectatomma opaciventre* (Hymenoptera: Formicidae) . Journal of Advanced Zoology 18 ( 2 ): 64 – 71 .

[R4] Antonialli-Junior WF Giannotti E . 2000 . Immature stages of workers of *Ectatomma edentatum* Roger, 1863 (Hymenoptera, Formicidae) . Revista Brasileira de Zoociências 2 ( 2 ): 105 – 113 .

[R5] Antonialli-Junior WF Giannotti E . 2001 . Nest architecture and population dynamics of the Ponerine ant *Ectatomma edentatum* (Hymenoptera, Formicidae) . Sociobiology 38 ( 3A ): 475 – 486 .

[R6] Antonialli-Junior WF Giannotti E . 2002 . Division of labor in *Ectatomma edentatum* (Hymenoptera, Formicidae) . Sociobiology 39 ( 1 ): 37 – 63 .

[R7] Antonialli-Junior WF Giannotti E . 2003 . Temporal polyethism in workers of the *Ectatomma edentatum* Roger (Hymenoptera, Formicidae) . Sociobiology 41 ( 1 ): 461 – 478 .

[R8] Antonialli-Junior WF Lima SM Andrade LHC Súarez R. 2007 . Comparative study of the cuticular hydrocarbon in queens, workers and males of *Ectatomma vizottoi* (Hymenoptera, Formicidae) by Fourier transform-infrared photoacoustic spectroscopy . Genetics and Molecular Research 6 ( 3 ): 492 – 499 . 17985301

[R9] Antonialli-Junior WF Súarez YR Izida T Andrade LHC Lima SM. 2008 . Intra and interspecific variation of cuticular hydrocarbon composition in two *Ectatomma* species (Hymenoptera: Formicidae) based on Fourier transform infrared photoacoustic spectroscopy . Genetics and Molecular Research 7 ( 2 ): 559 – 566 . 18752181 10.4238/vol7-2gmr454

[R10] Bagnères AG Blomquist GJ. 2010 . Site of synthesis, mechanism of transport and selective deposition of hydrocarbons. In: Blomquist GJ, Bagnères AG, Editors . Insect hydrocarbons: biology, biochemistry and chemical ecology . pp. 75 – 99 . Cambridge University Press.

[R11] Banschbach VS Herbers JM . 1996 . Complex colony structure in social insects: I: ecological determinants and genetic consequences . Evolution 50 ( 1 ): 285 – 297 . 28568882 10.1111/j.1558-5646.1996.tb04492.x

[R12] Blomquist GJ Tillman B Mpuru S Seybold SJ . 1998 . The cuticule and cuticular hydrocarbons of insects: structure, function, and biochemistry. In: Vander Meer RK, Breed MD, Winston ML, Espelie KE, Editors . Pheromone communication in social insect . pp. 35 – 54 . Westview Press .

[R13] Blomquist GJ Bagnères AG. 2010 . Introdution: history and overview of insects hydrocarbons. In: Blomquist GJ, Bagnères AG, Editors . Insect hydrocarbons: biology, biochemistry and chemical ecology . pp. 3 – 18 . Cambridge University Press .

[R14] Bolton B . 1994 . Identification Guide to the Ant Genera of the World . Harvard University Press .

[R15] Bolton AG Ward PS Naskrecki P . 2007 . Bolton’s catalogue of ants of the world . Harvard University Press .

[R16] Bonavita-Cougourdan A Clément JL Lange C. 1987 . Nestmate recognition: the role of cuticular hydrocarbons in the ant *Camponotus vagus* Scop . Journal of Entomological Science 22 : 1 – 10 .

[R17] Brady SG Schultz TR Fisher B Ward PS . 2006 . Evaluating alternative hypotheses for the early evolution and diversification of ants . Proceedings of the National Academy of Sciences of the United States of America 103 ( 48 ): 18. 172 – 18 .177. 10.1073/pnas.0605858103 PMC1838725 17079492

[R18] Breed MD Tony PA Bleuze J Denton SE . 1990 . Thievery, home ranges and nestmate recognition in *Ectatomma ruidum* . Oecologia 84 : 117 – 121 . 28312784 10.1007/BF00665604

[R19] Brown WL. Jr 1958 . Contributions toward a reclassification of the Formicidae. II. Tribe Ectatommini (Hymenoptera) . Bulletin of the Museum of Comparative Zoology 118 ( 5 ): 175 – 362 .

[R20] Clark PJ Evans FC . 1954 . Distance to nearest neighbour as measure of spatial relationship in populations . Ecology 34 : 445 – 453 .

[R21] Corrêa MM Fernandes WD Leal IR. 2006 . Diversidade de formigas epigéicas (Hymenoptera: Formicidae) em capões do Pantanal Sul Matogrossense: relações entre riqueza de espécies e complexidade estrutural da área . Neotropical Entomology 35 ( 6 ): 724 – 730 . 17273701 10.1590/s1519-566x2006000600002

[R22] Crozier RH Dix MW . 1979 . Analysis of two genetic models for the innate components of colony odour in social Hymenoptera . Behavioral Ecology and Sociobiology 4 : 217 – 224 .

[R23] Crozier RH Pamilo P . 1996 . Evolution of social insect colonies: Sex allocation and kin selection . Oxford University Press .

[R24] Cushman JH Martinsen GD Mazeroli AI . 1988 . Density and size dependent spacing of ant nests: evidence for intraspecific competition . Oecologia 77 : 522 – 525 . 28311272 10.1007/BF00377268

[R25] Cuvillier-Hot VV Cobb M Malosse C Peeters C . 2001 . Sex, age and ovarian activity affect cuticular hydrocarbons in *Diacamma ceylonense* , a queenless ant . Journal of Insect Physiology 47 : 485 – 493 . 11166313 10.1016/s0022-1910(00)00137-2

[R26] Dahbi A Lenoir A . 1998 . Nest separation and the dynamics of the “Gestalt” odour in the polydomous ant *Cataglyphis iberica* (Hymenoptera: Formicidae) . Behavioral Ecology and Sociobiology 42 : 349 – 355 .

[R27] Debout G Schatz B Elias M Mckey D . 2007 . Polydomy in ants: what we know, what we think we know, and what remains to be done . Biological Journal of the Linnean Society 90 : 19 – 348 .

[R28] Delabie JHC Alves HSR França VC Martins PTA Nascimento IC. 2007 . Biogeografia das formigas predadoras do gênero *Ectatomma* (Hymenoptera: Formicidae: Ectatomminae) no leste da Bahia e regiões vizinhas . Agrotrópica 19 : 13 – 20 .

[R29] Deslippe RJ Savolainen R . 1994 . Role of food supply in structuring a population of *Formica* ants . Journal of Animal Ecology 63 : 756 – 764 .

[R30] Deslippe RJ Savolainen R . 1995 . Mechanisms of competition in a guild of formicine ants . Oikos 72 : 67 – 73 .

[R31] Erdogmus GDVM . 2010 . A perda de área foliar e sua relação com o gênero Ectatomma (Formicidae: Ectatomminae) em uma comunidade de cerrado . Tese de Doutorado pela Universidade de São Paulo FFCLRP -Departamento de Biologia Programa de Pós-Graduação em Entomologia .

[R32] Fernández F. 1991 . Las hormigas cazadoras del genero *Ectatomma* (Hymenoptera: Formicidae) en Colombia . Caldasia 16 : 551 – 564 .

[R33] Forel A . 1874 . Les fourmis de la Suisse, Systématique. Notices anatomiques at physiologiques. Architecture. Distribution géographique. Nouvelles expériences et observations de moeurs, French Edition, University of Michigan Library .

[R34] Franz NM Wcislo WT. 2003 . Foraging behavior in two species of *Ectatomma* (Formicidae: Ponerinae): individual learning of orientation and timing . Journal of Insect Behavior 16 : 381 – 410 .

[R35] Greenslade PJM . 1975 . Dispersion and history of a population of the meat ant *Iridomyrmex purpureus* . Australian Journal of Zoology 23 : 495 – 510 .

[R36] Howard RW . 1993 . Cuticular hydrocarbons and chemical communication. In: Samuelson S, Nelson DR, Editors . Insect Lipids: Chemistry, Biochemistry and Biology . pp. 179 – 226 . University of Nebraska Press .

[R37] Howard RW Blomquist GJ . 2005 . Ecological, behavioral, and biochemical aspects of insect hydrocarbons . Annual Reviews of Entomology 50 : 371 – 93 . 10.1146/annurev.ento.50.071803.130359 15355247

[R38] Hölldobler B Wilson EO. 1977 . Weaver ants . Scientific American 237 ( 6 ): 146 – 154 .

[R39] Hölldobler B Wilson EO. 1990 . The Ants, first edition. The Belknap Press of Harvard University Press .

[R40] Jaffé K. 1993 . El mundo de las hormigas, first edition. Editora Equinoccio .

[R41] Jutsum AR Saunders TS Cherrett JM . 1979 . Intraspecific aggression in the leaf-cutting ant *Acromyrmex octospinosus* . Animal Behavior 27 : 839 – 844 .

[R42] Kugler C Brown Jr WL . 1982 . Revisionary and other studies on the ant genus *Ectatomma* , including the description of two new species . Search Agriculture 24 : 1 – 8 .

[R43] Lanan MC Dornhaus A Bronstein JL . 2011 . The function of polydomy: the ant *Crematogaster torosa* preferentially forms new nests near food sources and fortifies outstations . Behavior, Ecology and Sociobiology 65 : 959 – 968 .

[R44] Lapola DM Antonialli-Junior WF Giannotti E . 2003 . Arquitetura de ninho da formiga neotropical *Ectatomma brunneum* F. Smith, 1858 (Formicidae: Ponerinae) em ambientes alterados . Revista Brasileira de Zoociências 5 : 177 – 188 .

[R45] Laskis KO Tschinkel WR . 2009 . The seasonal natural history of the ant, *Dolichoderus mariae* , in northern Florida . Journal of Insect Science 9 : 2 . Available online: www.insectscience.org/9.2 19611227 10.1673/031.009.0201 PMC3011848

[R46] Lattke JE . 1994 . Phylogenetic relationships and classification of Ectatomminae ants (Hymenoptera: Formicidae) . Entomologica Scandinavica 25 : 105 – 119 .

[R47] Lattke JE . 2003 . Subfamilia Ponerinae. In: Fernández F, Editor . Introducción a las hormigas de la región Neotropical. pp. 261 – 276 . Instituto de Investigación de Recursos Biológicos Alexander von Humboldt .

[R48] Lenoir A Fresneau D Errard C Hefetz A . 1999 . Individuality and social representation concept . In: Information Processing in Social Insects . pp. 219 – 237 . Birkhäuser Verlag .

[R49] Levings SC Traniello AA . 1981 . Territoriality, nest dispersion, and community structure in ants . Psyche 88 : 265 – 319 .

[R50] Liang D Silverman J . 2000 . “You are what you eat”: Diet modifies cuticular hydrocarbons and nestmate recognition in the Argentine ant, *Linepithema humile* . Naturwissenshaften 87 : 412 – 416 . 10.1007/s001140050752 11091966

[R51] Locher GD Giannotti E Tofolo VC . 2009 . Brood care behavior in *Ectatomma brunneum* (Hymenoptera, Formicidae, Ectatomminae) under laboratory conditions . Sociobiology 54 : 573 – 587 .

[R52] Marinho CGS Zanetti R Delabie JHC Schlindwein MNE Ramos LS . 2002 . Diversidade de formigas (Hymenoptera: Formicidae) da serrapilheira em eucaliptais (Myrtaceae) e área de cerrado de Minas Gerais . Neotropical Entomology 31 ( 2 ): 187 – 195 .

[R53] Marques GDV Del-Claro K. 2006 . The ant fauna in a cerrado area: the influence of vegetation structure and seasonality (Hymenoptera: Formicidae) . Sociobiology 47 : 235 – 252 .

[R54] Meunier J Delémont O Lucas C. 2011 . Recognition in Ants: Social Origin Matters . PLOS One 6 ( 5 ): 1 – 6 . 10.1371/journal.pone.0019347 PMC3087756 21573235

[R55] Moreau CS Bell CD Vila R Archibald SB Pierce NE . 2006 . Phylogeny of the Ants: Diversification in the Age of Angiosperms . Science 12 : 101 – 104 . 10.1126/science.1124891 16601190

[R56] Nowbahari E Lenoir A Clement JL Lange C Bagneres AG Joulie C . 1990 . Individual, geographical and experimental variation of cuticular hydrocarbons of the ant *Cataglyphis cursor* (Hymenoptera: Formicidae): their use in nest and subspecies recognition . Biochemical Systematics and Ecology 18 : 63 – 73 .

[R57] Oliveira PS Brandão CRF. 1991 . The ant community associated with extrafloral nectaries in the Brazilian cerrados. In Huxley CR, Cutler DF, Editors . Ant-plant interactions . pp. 199 – 212 . Oxford University Press .

[R58] Oliveira PS Del-Claro K. 2005 . Multitrophic Interactions in a Neotropical savanna: ant-hemipteran systems, associated insect herbivores, and a host plant. In: Burslem DFRP, Pinard MA, Hartley SE, Editors . Biotic interactions in the tropics . pp. 414 – 440 . Cambridge University Press .

[R59] Oliveira PS Freitas AVL . 2004 . Ant-plant-herbivore interactions in the Neotropical cerrado savanna . Naturwissenschaften 91 : 557 – 570 . 15551026 10.1007/s00114-004-0585-x

[R60] Paiva RVS Brandão CRF. 1989 . Estudos sobre a organização social de *Ectatomma permagnum* Forel, 1908 (Hymenoptera: Formicidae) . Revista Brasileira de Biologia 49 : 783 – 792 .

[R61] Pedersen JS Boomsma JJ . 1999 . Multiple paternity in social Hymenoptera: estimating the effective mate number in single-double mating populations . Molecular Ecology 8 ( 4 ): 577 – 587 .

[R62] Pie MR . 2004 . Foraging ecology and behaviour of the Ponerinae ant *Ectatomma opaciventre* Roger in a Brazilian savanna . Journal of Natural History 38 : 717 – 729 .

[R63] Pratt SC . 1989 . Recruitment and other communication behavior in the Ponerine ant *Ectatomma ruidum* . Ethology 81 : 313 – 331 .

[R64] Provost E . 1989 . Social environmental factors influencing mutual recognition of individuals in the ant *Leptothorax lichtensteini* (Hymenoptera: Formicidae) Bondr . Behavioral Processes 18 : 35 – 39 . 10.1016/S0376-6357(89)80004-X 24897665

[R65] Redolfi I Ruano F Tinaut A Pascual F Campos M . 2005 . Ant nests spatial distribution and temporary permanence in olive orchards at Granada, Spain . Ecología Aplicada 4 ( 1,2 ): 71 – 76 .

[R66] Ryti RT Case TJ . 1984 . Spatial arrangement and diet overlap between colonies of desert ants . Oecologia 62 : 401 – 404 . 28310895 10.1007/BF00384274

[R67] Ryti RT Case TJ . 1986 . Overdispersion of ant colonies: a test of hypotheses . Oecologia 69 : 446 – 453 . 28311347 10.1007/BF00377067

[R68] Santos GM De M Delabie JHC Resende JJ. 1999 . Caracterização da mirmecofauna (Hymenoptera: Formicidae) associada à vegetação periférica de inselbergs (caatinga-arbórea-estacionalsemidecídua) em Itatim -Bahia -Brasil . Sitientibus 20 : 33 – 43 .

[R69] Scott-Santos CP Esteves FA Brandão CRF. 2008 . Catalogue of "poneromorph" ant type specimens (Hymenoptera, Formicidae) deposited in the Museu de Zoologia da Universidade de São Paulo, Brazil . Papéis Avulsos de Zoologia 48 ( 11 ): 75 – 88 .

[R70] Seifert B . 2010 . Intranidal mating, gyne polymorphism, polygyny, and supercoloniality as factors for sympatric and parapatric speciation in ants . Ecological Entomology 35 : 33 – 40 .

[R71] Silvestre R Silva RR Da . 2001 . Guildas de formigas da Estação Ecológica Jataí, Luiz Antônio-SP – sugestões para aplicação do modelo de guildas como bio-indicadores ambientais . Biotemas 14 ( 1 ): 37 – 69 .

[R72] Singer TL . 1998 . Roles of hydrocarbons in the recognition systems of insects . American Zoologist 38 : 394 – 405 .

[R73] Step E . 1924 . Go to the Ant . Hutchinson and Co .

[R74] Sturgis S Gordon DM . 2012 . Nestmate recognition in ants (Hymenoptera, Formicidae): a review . Myrmecological News 16 : 101 – 110 .

[R75] Tofolo VC . 2011 . Dinâmica populacional, forrageamento e exposição de operárias de Ectatomma opaciventre (Hymenoptera: Formicidae: Ectatomminae) a iscas formicidas contendo sulfluramida, fipronil e clorpirifós . Tese de Doutorado em Ciencias Biologicas (Zoologia) , Universidade Estadual Paulista Júlio de Mesquita Filho , UNESP, Brasil .

[R76] Tofolo VC Giannotti E Moleiro HR Simões MR. 2011 . Diet and Spatial Pattern of Foraging in *Ectatomma opaciventre* (Hymenoptera: Formicidae) in an Anthropic Area . Sociobiology 58 : 607 – 620 .

[R77] Tofolo VC Giannotti E . 2005 . Population dynamics of *Ectatomma brunneum* (Hymenoptera; Formicidae) under laboratory conditions . Sociobiology 46 ( 3 ): 627 – 636 .

[R78] Tofolo VC Giannotti E Pizano MA . 2010 . Foraging behavior and mortality of *Ectatomma brunneum* (Hymenoptera, Formicidae) in simultaneous exposure to ant baits and conventional diet in laboratory . Sociobiology 55 : 599 – 611 .

[R79] Tofolo VC Albino E Nomura E Fowler HG . 2006 . Predatory behavior of *Ectatomma brunneum* (Hymenoptera, Formicidae, Ectatomminae) under laboratory conditions . Revista de Etologia 8 supl: 105 – 106 .

[R80] Traniello AA . 1989 . Foraging strategies of ants . Annual Review of Entomology 34 : 191 – 210 .

[R81] Valentin JL . 2000 . Ecologia Numérica: uma introdução à análise multivariada de dados ecológicos, first edition. Editora Interciência .

[R82] van Zweden JS D’Ettorre P. 2010 . Nestmate recognition in social insects and the role of hydrocarbons. In: Blomquist GJ, Bagnères AG, Editors . Insect hydrocarbons: biology, biochemistry and chemical ecology . pp. 222 – 243 . Cambridge University Press .

[R83] Vieira AS Antonialli-Junior WF. 2006 . Populational fluctuation and nest architecture of *Ectatomma brunneum* (Hymenoptera, Formicidae) in remaining areas of pasture, Dourados -MS, Brasil . Sociobiology 47 ( 1 ): 275 – 287 .

[R84] Vieira AS Antonialli-Junior WF Fernandes WD . 2007 . Modelo arquitetônico de ninhos da formiga *Ectatomma vizottoi* Almeida (Hymenoptera, Formicidae) . Revista Brasileira de Entomologia 51 : 489 – 493 .

[R85] Vieira AS Antonialli-Junior WF Fernandes WD Tofolo VC Giannotti E . 2009 . Description of the immature and adult stages of *Ectatomma vizottoi* (Formicidae: Ectatomminae) . Sociobiology 53 : 27 – 38 .

[R86] Vieira AS Bueno OC Camargo-Mathias MI. 2010 . The functional morphology of the metapleural gland of the leaf-cutting ant *Atta laevigata* (Formicidae: Attini) . Micron 41 : 149 – 157 . 19926295 10.1016/j.micron.2009.08.012

[R87] Wheeler D.E. . 1986 . *Ectatomma tuberculatum* foraging biology and association with *Crematogaster* (Hymenoptera Formicidae) . Annals of the Entomological Society of America 79 : 300 – 303 .

[R88] Wilson EO Brown Jr WL . 1984 . Behavior of the cryptobiotic predaceous ant *Eurhopalothrix heliscata* , n. sp. (Hymenoptera: Formicidae: Basicerotini) . Insectes Sociaux 31 : 408 – 428 .

[R89] Zinck L Hora RR Chaline N Jaisson P . 2008 . Low intraspecific aggression level in the polydomous and facultative polygynous ant *Ectatomma tuberculatum* . Entomologia Experimentalis et Applicata 126 : 211 – 216 .

